# Gearing up to handle the mosaic nature of life in the quest for orthologs

**DOI:** 10.1093/bioinformatics/btx542

**Published:** 2017-08-30

**Authors:** Kristoffer Forslund, Cecile Pereira, Salvador Capella-Gutierrez, Alan Sousa da Silva, Adrian Altenhoff, Jaime Huerta-Cepas, Matthieu Muffato, Mateus Patricio, Klaas Vandepoele, Ingo Ebersberger, Judith Blake, Jesualdo Tomás Fernández Breis, The Quest for Orthologs Consortium, Brigitte Boeckmann, Toni Gabaldón, Erik Sonnhammer, Christophe Dessimoz, Suzanna Lewis, Quest for Orthologs Consortium, Adrian Altenhoff, Carla Bello, Judith Blake, Brigitte Boeckmann, Sébastien Briois, Salvador Capella-Gutierrez, Edward Chalstrey, Hirokazu Chiba, Oscar Conchillo-Solé, Vincent Daubin, Todd DeLuca, Christophe Dessimoz, Jean-Francois Dufayard, Dannie Durand, Ingo Ebersberger, Jesualdo Tomás Fernández-Breis, Kristoffer Forslund, Natasha Glover, Alexander Hauser, Davide Heller, Jaime Huerta-Cepas, Mateusz Kaduk, Jan Koch, Eugene V Koonin, Evgenia Kriventseva, Shigehiro Kuraku, Odile Lecompte, Olivier Lespinet, Jeremy Levy, Suzanna Lewis, Benjamin Liebeskind, Benjamin Linard, Marina Marcet-Houben, Maria Martin, Claire McWhite, Sergei Mekhedov, Sebastien Moretti, Matthieu Muffato, Steven Müller, El-Mabrouk Nadia, Cédric Notredame, Mateus Patricio, Simon Penel, Cécile Pereira, Ivana Pilizota, Henning Redestig, Marc Robinson-Rechavi, Fabian Schreiber, Kimmen Sjölander, Nives Škunca, Erik Sonnhammer, Alan Sousa da Silva, Martin Steinegger, Damian Szklarczyk, Paul Thomas, Ernst Thuer, Clément Train, Ikuo Uchiyama, Klaas Vandepoele, Lucas Wittwer, Ioannis Xenarios, Bethan Yates, Evgeny Zdobnov, Robert M Waterhouse

**Affiliations:** 1 Structural and Computational Biology Unit, European Molecular Biology Laboratory, Heidelberg, Germany; 2 Microbiology and Cell Science Department, Institute for Food and Agricultural Sciences, University of Florida at Gainesville, Gainesville, FL, USA; 3 Laboratoire de Recherche en Informatique (LRI); 4 Institute for Integrative Biology of the Cell (I2BC), CEA, CNRS, Univ. Paris‐Sud, Université Paris‐Saclay, Gif‐sur‐Yvette cedex, France; 5 Spanish National Bioinformatics Institute (INB), Spanish National Cancer Research Centre, Madrid, Spain; 6 European Molecular Biology Laboratory, European Bioinformatics Institute, Wellcome Genome Campus, Hinxton, UK; 7 Department of Computer Science, ETH Zurich, Zurich, Switzerland; 8 Computational Biochemistry Research Group, Swiss Institute of Bioinformatics (SIB), Zurich, Switzerland; 9 VIB & Department of Plant Biotechnology and Bioinformatics, Center for Plant Systems Biology, Ghent University, Ghent, Belgium; 10 Applied Bioinformatics, Goethe-Universitet Frankfurt, Frankfurt, Germany; 11 Senckenberg Climate and Research Center Frankfurt (BIK-F), Frankfurt, Germany; 12 Jackson Laboratory, Bar Harbor, ME, USA; 13 Faculty of Computer Science, University of Murcia, IMIB-Arrixaca, Murcia, Spain; 14 Swiss-Prot Group, Swiss Institute of Bioinformatics (SIB), Geneva, Switzerland; 15 Bioinformatics and Genomics Programme, Centre for Genomic Regulation (CRG), Barcelona Institute of Science and Technology (BIST), Barcelona, Spain; 16 Universitat Pompeu Fabra (UPF), Barcelona, Spain; 17 Institució Catalana de Recerca I Estudis Avançats (ICREA), Barcelona, Spain; 18 Science for Life Laboratory, Department of Biochemistry and Biophysics, Stockholm Bioinformatics Center, Stockholm University, Solna, Sweden; 19 Center for Integrative Genomics, University of Lausanne, Lausanne, Switzerland; 20 Department of Ecology and Evolution, University of Lausanne, Lausanne, Switzerland; 21 Department of Genetics, Evolution, and Environment, University College London, London, UK; 22 Department Computer Science, University College London, London, UK; 23 Swiss Institute of Bioinformatics, CH-1015 Lausanne, Switzerland; 24 Environmental Genomics and Systems Biology, Lawrence Berkeley National Laboratory, Berkeley, CA, USA

## Abstract

**Summary:** The Quest for Orthologs (QfO) is an open collaboration framework for experts in comparative phylogenomics and related research areas who have an interest in highly accurate orthology predictions and their applications. We here report highlights and discussion points from the QfO meeting 2015 held in Barcelona. Achievements in recent years have established a basis to support developments for improved orthology prediction and to explore new approaches. Central to the QfO effort is proper benchmarking of methods and services, as well as design of standardized datasets and standardized formats to allow sharing and comparison of results. Simultaneously, analysis pipelines have been improved, evaluated and adapted to handle large datasets. All this would not have occurred without the long-term collaboration of Consortium members. Meeting regularly to review and coordinate complementary activities from a broad spectrum of innovative researchers clearly benefits the community. Highlights of the meeting include addressing sources of and legitimacy of disagreements between orthology calls, the context dependency of orthology definitions, special challenges encountered when analyzing very anciently rooted orthologies, orthology in the light of whole-genome duplications, and the concept of orthologous versus paralogous relationships at different levels, including domain-level orthology. Furthermore, particular needs for different applications (e.g. plant genomics, ancient gene families and others) and the infrastructure for making orthology inferences available (e.g. interfaces with model organism databases) were discussed, with several ongoing efforts that are expected to be reported on during the upcoming 2017 QfO meeting.

## 1 Introduction

Orthology is defined as the relationship between a pair of sequences separated through a species divergence event from their most recent common ancestor (Fitch, 1970). Paralogy, in contrast, follows sequence duplication events resulting in eventually divergent sequences within the same (ancestral) genome. Given our understanding of evolutionary forces, we expect a relatively higher conservation of function in orthologs than in paralogs. This distinction is crucial when transferring knowledge from assays and analyses between species, such as selecting the correct orthologous target for knockout in an experimental system. The distinction is further needed in exploratory evolutionary analysis, because it is primarily orthologs that are expected to follow the phylogeny of their species. The definition is made more complicated still in that subsequent duplications after a speciation event results in several sequences in a species all being (in-)paralogous with each other and (co-)orthologous to their counterparts in another species. From the time that sequence data first became available, multiple independent efforts have striven to develop methods for identifying orthologs and paralogs. This has resulted in a number of publicly available and widely used resources.

The Quest for Orthologs (QfO, http://questfororthologs.org) initiative has so far organized four biennial gatherings (http://questfororthologs.org/meetings) for the research community with an interest in orthology determinations—including both developers and application users—with the aim of sharing experiences, establishing metrics, improving data exchange and comparability, as well as developing joint strategies for advancing the field. Starting with the 2009 meeting in Hinxton (Cambridge, UK), the work centered on establishing standardized reference proteomes to benchmark different tools, developing frameworks to facilitate data exchange (e.g. OrthoXML and SeqXML, Schmitt *et al.*, 2011), and establishing collaborative working groups to keep advancing on these and other issues. While the adoption of standard formats took time, most orthology resources reported recently to provide support for OrthoXML (Sonnhammer *et al.*, 2014), thus paving the way for applications such as joint benchmarking or consensus meta-analysis servers for orthology assessment (Pereira *et al.*, 2014).

The second QfO meeting in 2011, also in Hinxton (Cambridge, UK), discussed the necessity of a common framework for well-curated and well-established biological datasets. Indeed, stable versions of reference proteomes representing species from a broad taxonomic spread would greatly facilitate comparison of the results from different orthology analysis pipelines and disentangle effects of input data choices (genome versions, gene calls, taxonomic range, …) from that of the algorithms. Since then, an increasing number of researchers and tool developers have joined the working groups in charge of the coordination and maintenance of those resources (e.g. a curated set of reference proteomes). Additionally, since orthology fundamentally relates nested histories (e.g. that of proteins) to their host histories (e.g. those of species), in many cases an accurate consensus species phylogeny is needed. Because of this mutual dependency, a QfO species tree working group, along with representatives from the community of researchers working on the ‘Tree of Life’ problem, was initiated in 2013 to review the congruence of species classifications. This work resulted in an overview of consensus and disagreement with regards to species phylogeny on the medium scale (Boeckmann *et al.*, 2015), and an agreed upon species-level phylogeny for joint QfO analysis.

Orthology inference methods must all cope with choosing among multiple complex evolutionary scenarios, each with a risk of conjuring up a dubious assignment. During the third Quest for Orthologs meeting in 2013 in Lausanne, Switzerland, there was a growing recognition of the need for careful benchmarking to profile and assess the performance of orthology inference methods. One theme of this discussion was how to design such benchmarks. Starting from a review of the work independently carried out by several teams in developing orthology benchmarks and curated gold standard datasets, a QfO working group began designing a shared resource for comparative evaluation of orthology calls. This online service assists developers as they each strive to improve their respective algorithms and resources, and offers guidance for users in selecting the most appropriate method given their particular application’s goal. This tool has now been published (Altenhoff *et al.*, 2016) and is available online (http://orthology.benchmarkservice.org). This service also functions as an up-to-date reference to the benchmarked tools (see http://orthology.benchmarkservice.org/projects).

In 2015, the fourth Quest for Orthologs meeting was held in Barcelona. It was widely attended in terms of the range of participating orthology inference specialists, including American, European and Asian teams, as well as with regards to the active participation of industrial researchers with interests in particular applications of orthology. The present report highlights the main points of discussion during that meeting, and aims to place those discussions within the larger context of the field of orthology inference; including where that field may be heading and what the challenges are that we must meet in order to get there.

## 2 Meeting highlights

As a wider and wider taxonomic diversity of genomic information becomes available, the challenges and opportunities for resolving orthologs, relative to very ancient ancestral species, increases proportionately. The further back we look, the more challenging it becomes to reconstruct evolution accurately from sequence data. Improvements to current strategies were presented at this meeting that help overcome some of these difficulties.

### 2.1 Orthology conjecture and evolutionarily deep orthologies

Orthology as the central concept of evolutionary genomics was the theme of the keynote speaker, Eugene Koonin (NCBI/NIH, USA). Orthologous (as opposed to paralogous) genes are often assumed to share the highest sequence similarity, the highest structural conservation, as well as retaining a common ancestral function. This has been termed the ‘orthology conjecture’ (Nehrt *et al.*, 2011). Although these implicit corollaries are not part of the orthology definition itself, they seem to hold for a large fraction of orthologous genes in large-scale comparative genomics studies (discussed in Gabaldón and Koonin, 2013). Evolution, however, is a highly complex process. Indeed, the prediction of orthologous genes from closely related organisms is straightforward, but only a small fraction of highly conserved genes are predicted to be orthologous between genomes from distantly related species, because the increasing accumulation of evolutionary events in a gene’s history obfuscates these relationships. In particular, the diversification of domain architectures in protein-coding genes in many cases accounts for the relative lack of predicted orthologs. Deep phylogenies are therefore best studied on individual orthologous protein domains rather than on full-length orthologous genes. In summary, the simplifying corollaries of the ‘orthology conjecture’ will need to be revised as the model of evolution is refined.

### 2.2 Orthologous evolution at different scales

**Fig. 1:**
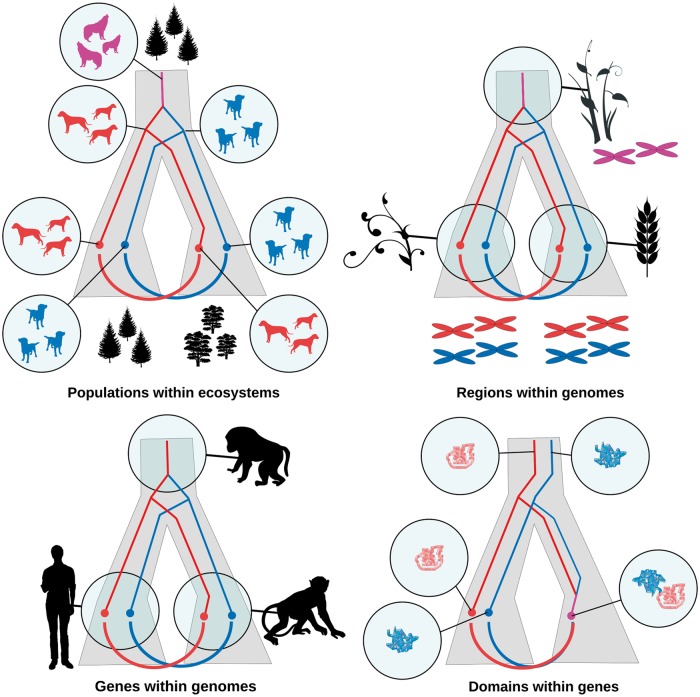
Nested Evolutionary Histories: Containing (e.g. species) trees (grey area) with nested (e.g. gene) trees (red/blue) inside. Semicircles denote orthologous or orthology-like relations. On the top level (top left panel), subpopulations unique to a particular ecological niche are nested within populations, and within subpopulations in turn, the histories of individual genealogies are nested. In this sense, niche segregation within a site is a form of duplication event, whereas migration to novel habitats correspond to speciation events at this level. In addition, genes lie within genomes that are subject to whole-genome or chromosome-scale duplication events (top right panel, bottom left panel), which strongly influences genome evolution and involve discordances between the histories of components and wholes, and protein domains within genes may reshuffle over evolutionary time (bottom right panel), complicating the use of full-length sequences in orthology inference (Color version of this figure is available at *Bioinformatics* online.)

Orthology relates sequence features in a nested history (i.e. the evolutionary scenarios joining together extant gene sequences) to their surrounding ‘host’ history (i.e. the evolution of the species within which these are/were found) as outlined in Figure 1. Similar relationships can be identified on multiple levels, a point highlighted by several participants. Below the conventional level of contrasting gene versus species phylogenies, orthology analysis can be conducted at the level of homology/ohnology-type evolution, that is, large-scale paralogies resulting from tandem, segmental or whole genome duplication. Likewise, domain-level analyses can account for evolutionary events that shuffle domains around within genes, causing hybrid histories. Cedric Notredame presented a method that incorporates key protein structural elements to improve upon purely sequence-based multiple sequence alignment methods (Chang *et al.*, 2015). Furthermore, similar relationships exist by analogy at the level of (sub-)populations within niches or ecosystems, with migration resembling speciation and niche diversification within a site duplication in this regard.

Additionally, drawing on discussions in previous meetings, the context dependency of the definition of orthology was highlighted, wherein orthologous pairs or groups are defined as extant sequences descended from the same ancestral sequence. Pair-wise methods naturally require such ancestral sequences to exist specifically in the last common ancestor (LCA) species of the genomes where the extant pair members are found, whereas, to be meaningful, multi-species/group methods require an LCA genome to be explicitly specified, as well as allowing for subsequent gene duplications to introduce some cases of (in-)paralogy (Sonnhammer and Koonin, 2002) between group members. It was particularly noted that unless such contexts are properly taken into account, applications drawing on multi-species orthology groups may yield counterintuitive results. A strong recommendation therefore emerged to ensure that the ancestral species relative to which each orthologous group is defined is clearly specified in resources and applications.

### 2.3 Working group updates

Most progress achieved by the QfO aside from the meetings themselves has taken place in working groups for special interests, which regularly communicate between meetings. These thus far have included a species tree resource working group, an orthology benchmarking working group, and a working group for construction of novel orthology utility tools. To ensure greater transparency and accessibility to orthology researchers who may wish to participate, contact information for all working groups are available at http://questfororthologs.org/working_groups.

### 2.4 First steps in synchronizing orthology with biological systematics

Tree-based orthology inference presumes knowledge of the species tree. For this reason, the Quest for Orthologs species tree working group surveyed the classifications of 147 organisms of interest to the QfO, including the most well-studied model organisms, and reported areas of congruence and particularly high incongruence in the outcomes of different evolutionary history reconstruction efforts (Boeckmann *et al*, 2015). Six large-scale and well-used trees of life (ToL) and species classifications were analyzed, among them the National Center for Biotechnology Information (NCBI) taxonomy (Federhen *et al.*, 2012), the Open Tree of Life (Hinchliff *et al.*, 2015) and the 16S ribosomal RNA (rRNA) database (Yilmaz *et al.*, 2013). Comparing the species phylogenies to a consensus species tree model of the 147 taxa revealed topological discordance and ambiguity for about 40% of all clades, notably in both ancient and recent regions of the tree. Large-scale mapping of gene trees to the consensus species trees coincides with the level of observed incongruence between species classifications. In particular, incomplete lineage sorting, hybridization and allopolyploidy interfere with species tree-aware orthology prediction for eukaryotes, and horizontal gene transfer is likely the main reason for the species tree discordance seen in the prokaryotic domains of life. Alternative species tree topologies are thus a valuable source of information for more detailed studies on genome, lineage and species evolution (Hahn and Nakhleh, 2015). Applying such knowledge can help improve sequence-based phylogenies and explain non-tree-like structures in the ToL. Caution is advised when predicting orthologs based on orthology-inferred species trees, and those ToLs inferred with alternative, complementary methods are therefore of particular importance to the QfO (Boeckmann *et al.*, 2015).

### 2.5 Common standards and representations

The previously introduced OrthoXML standard for representing orthology calls, as stated, is provided by successively more resources, which was highlighted in several presentations. Plans were discussed further in making use of a common framework (e.g. a mini-API for web services providing OrthoXML calls given a sufficiently context-aware input set) both for joint benchmarking and for consensus orthology assessments, as a possible future QfO-provided resource. Discussion during the meeting also highlighted the potential of applying semantic web principles and concepts to produce RDF implementations of orthology resources. Progress on the development of the Orthology Ontology (Fernández-Breis *et al.*, 2016) and its application to generate RDF versions of OrthoXML resources were presented. While more work remains to be done before such a system is fully adapted and in broad use among orthology call providers, the operations of a working group for development of orthology utilities has led to steady progress addressing specific technical issues and research questions.

## 3 Algorithmic advances

### 3.1 Real and apparent discrepancies between conclusions from different methods

With recent developments of the joint Quest for Orthologs benchmarking datasets and tools, this meeting renewed discussions on the extent to which, and the reasons why, different methods may produce incompatible orthologous and paralogous relationship calls. The first publication of benchmark results (Altenhoff *et al.*, 2016) provides a large-scale quantification of such discrepancies also accessible online (ttp://orthology.benchmarkservice.org/). Paul D. Thomas (University of Southern California, USA) pointed out that differences in *homology* clustering (e.g. alignment score cutoffs, alignment coverage requirements, or cluster tightness), could have a substantial impact on the downstream orthology inference independent of other more unique aspects of a method—the extent of which has yet to be investigated. To improve homology inference, Mateus Patricio (EMBL-EBI, UK) presented a new library of Hidden Markov Model (HMM) profiles built upon the PANTHER (Mi *et al.*, 2015) and TreeFam (Schreiber *et al.*, 2014) databases and complemented with new profiles inferred from sequences that were not covered by any of these libraries. The library covers all the eukaryotes, including vertebrates and non-vertebrates, and is available for download on the Ensembl FTP site (ftp://ftp.ensembl.org/pub/current_compara). The most recent version of PANTHER was also improved following feedback from this work. Furthermore, to go one step further and standardize the input of tree-based orthology methods, Thomas and Matthieu Muffato (EMBL-EBI, UK) proposed to work together on a common resource for protein family trees.

The parallel approach to these efforts to improve each of the methods is to combine their output in order to obtain a consensus. For example MARIO (Pereira *et al.*, 2014) presented by Cécile Pereira (Paris-Sud University, France), combines ortholog predictions from multiple methods, input in the standard orthoXML format, with the aim of obtaining more reliable orthology calls. This method was used in to perform the most recent update of FUNGIpath (Grossetête *et al.*, 2010), a database for comparative analysis of fungal metabolism.

### 3.2 Big data and scalability

One issue previously identified as a growing concern is the vast scale that high-throughput sequencing of genomes brings to orthology analysis (Sonnhammer *et al.*, 2014). Since the 2013 meeting, several participants highlighted their recent technical developments to improve scalability. For instance, several resources, including TreeFam and eggNOG (Huerta-Cepas *et al.*, 2016), now represent orthologous groups through Hidden Markov Model sequence representations which allow identification of novel members of families without the compute-intensive all-versus-all sequence comparisons. HMMs provide a more stable annotation of proteins since each sequence is classified independently from the others, as opposed to the all-vs-all methods that tend to define families globally and are more sensitive to changes in the input graph. Hieranoid (Schreiber and Sonnhammer, 2013) achieves linear scaling to the number of species by aggregating orthologs along a species guide tree. Another approach dealing with a similar problem was presented in the form of the MMseqs many-versus-many sequence comparison software (Hauser *et al.*, 2016). Still other approaches were suggested, such as making use of the nested nature of taxonomy and the transitive property of homology for more rapid identification of such sequences.

### 3.3 Inclusion of additional biological information

The meeting also featured several algorithmic contributions. Of particular note was work presented by Benjamin Liebeskind and Claire McWhite (University of Texas at Austin, USA) that leveraged conserved protein networks to better discern between paralogs and orthologs, focusing on networks that are involved in or associated with human diseases (Liebeskind, 2016; McWhite *et al.*, 2015). Similarly Klaas Vandepoele (University of Ghent – VIB, Belgium) compares gene expression level commonalities across different plant species to gain additional insight for correctly identifying functionally conserved (co-) orthologs showing conserved spatial-temporal expression (Movahedi *et al.*, 2012; Tzfadia *et al.*, 2016).

### 3.4 Applications

Increasing participation in QfO by researchers within industry and academia, who are leveraging orthology assertions in their applications, highlighted the need to consider a broad variety of application requirements. Consumers of orthology resources perceive the impact of alternative approaches yielding different orthologs calls and thus benefit from better characterization of the inherent tradeoffs between such approaches.

### 3.5 Orthology work within different taxonomic clades

The biology of different taxonomic groups (animals, plants, fungi, bacteria, …) is unique and therefore they each have different uses and encounter different obstacles in using ortholog calls. For example, in plants multiple whole genome duplications confound genomic assembly algorithms and thus researchers leverage ortholog calls to improving the coverage of their assemblies, as Klaas Vandepoele illustrated with the PLAZA comparative genomics platform (Proost *et al.*, 2014), another such application being OrthoFiller (http://biorxiv.org/content/early/2017/01/05/098566). There are increasing needs for plant-specific resources both for ecology and for advancing crop science, with major commercial actors such as Bayer and Syngenta therefore also participating in the QfO. These needs include ascertaining comparative taxonomic ranges that adequately span plant variability, and accounting for special challenges in plant genome analysis such as widespread polyploidization, repetitive elements and sometimes very large genome sizes. OMA (Altenhoff *et al.*, 2014) already includes plants, but as a direct result of discussions at the meeting, a QfO working group will seek to augment the QfO reference proteomes to allow better representation of certain species groups, in particular for plants, in anticipation of the next meeting in 2017.

### 3.6 Facilitating functional inference through orthology

The study of orthology is essentially research into evolutionary history, including, in part, extending our understanding of the evolution of functional capabilities. With a better understanding of orthology and paralogy, the function of proteins in less experimentally tractable systems can be more reliably inferred from those in which laboratory experiments can be more readily performed. Several participants highlighted the challenges encountered in functional inference through orthology. For example, the impact of whole genome duplication event was discussed in talks by Shigehiro Kuraku (RIKEN CLST, Japan) and Klaas Vandepoele. Part of the response may be found in improving orthology-based methods for protein function annotation. Nives Škunca (ETH Zurich, Switzerland) reported an improvement in Gene Ontology inference when propagating annotations across Hierarchical Orthologous Groups (HOGs) and not merely across groups of strict orthologs (Altenhoff *et al.*, 2015)—thus highlighting the benefits of also considering certain paralogous relationships. Moreover, different groups provided updates on the phylogenetic-based propagation of functional annotations, including Suzanna Lewis (Lawrence Berkeley National Laboratories, USA) presenting the Gene Ontology Consortium’s use of PANTHER and PAINT (Gaudet *et al.*, 2011) for manually annotating protein family trees, or Evgenia Kriventseva (University of Geneva, Switzerland) describing OrthoDB’s integration of functional information from UniProt and InterPro (Kriventseva *et al.*, 2014). Tools are also in the works for using orthology calls to annotate the function of custom sequences (e.g. eggNOG-mapper: http://biorxiv.org/content/early/2016/09/22/076331).

### 3.7 Model systems for insights into human disease mechanisms

Until recently the various projects dealing with important model organisms, such as ZFIN, FlyBase, MGI, SGD, WormBase and others, independently made their own choices with regards to which orthology prediction strategy they would use. End users of these resources perceived the impact of these alternative choices in that the reciprocity of ortholog calls across the model organism resources was unreliable. This lack of convergence on a common approach among the model organisms is particularly deleterious when orthology relationships are used to transfer evolutionarily inherited characteristics, such as gene function, or when they are used to select the most appropriate model system for the analysis of a particular disease. Despite the fact that current approaches by the model organism resources are less than ideal, the situation is improving. From a research infrastructure perspective, there is the recent formation of the Alliance for Genomic Resources (AGR, see http://www.alliancegenome.org/), which, among other objectives, will be unifying the model organisms into a single resource using the same strategies for ortholog calling in every case. Paul Thomas and Suzanna Lewis are engaging with AGR as liaisons to the QfO Consortium members.

## 4 Outlook: the mosaic nature of life and its impact on evolutionary studies

Alongside practical challenges such as scale and redundancy of datasets, a common trend in the discussions this year of challenges faced within orthology analysis stems from that living and evolving systems, while describable as having a tree-like history at one level, often contain subsystems with conflicting histories. Species, seen as collectives of organism lineages, will contain divergent component lineages and will experience, through migrations or habitat shifts, eventual separation into new species. Genomes, on one level understandable through their species history, will contain internal paralogies resulting from duplication of some or all regions within them, along with effects of gene loss or conversion. Genes can lose domains or gain domains from a different origin. In each case, the central problem is that not all subsystems reflect the history of the whole (Fig. 1). Since molecular evolution analysis hinges on the use of subsystems for e.g. marker genes in order to understand the evolution of the whole species, this is a challenge to take seriously. At such distances where structural similarity chiefly is what remains, orthology analyses will often in practice be the identification of orthologous domains rather than orthologous genes, though the extent to which this imposes limitations on the interpretations of results is something that needs to be explored further. At previous QfO meetings it has been noted that domain-level orthology often disagrees with the results of whole gene-level analyses. Both approaches have advantages and disadvantages, yet they are not easily reconcilable. As the scope of the analyses attempted widens in terms of taxa considered, it also deepens to more ancient divergence events, marking the need to achieve a comprehensive understanding of how to work with such domain-level mosaics as an upcoming goal of the orthology community.

Taken together, these challenges, alongside previously recognized issues such as the need for standardization, tools for fair benchmarking of algorithms, and sensible strategies to handle the rapid growth of the number of sequenced genomes for analysis, highlight the utility for community-wide coordination efforts as has been achieved through the Quest for Orthologs meetings and activities.
